# Dr. Frost and Poliomyelitis

**Published:** 1911-01

**Authors:** 


**A Monthly Review of Medicine and Surgery**

*EDITOR*

WILLIAM WARREN POTTER, M. D.

All communications, whether of a literary or business nature, books for review and exchanges, should be addressed to the editor, 238 DELAWARE AVENUE, BUFFALO, N.Y.

VOL. LXVI. JANUARY, 1911. No. 6

### Dr. Frost and Poliomyelitis

IN our editorial notice in the JOURNAL last month of the sanitary conference recently held in Buffalo, we adverted to the fact that several distinguished men from outside the state were present and lent their valuable counsel toward its perfection, among whom we mentioned Dr. W. H. Frost, of the marine hospital service, Washington, D. C.

Dr. Frost favored the conference with an interesting paper, entitled "The field investigation of epidemic poliomyelitis; what the health officer can do toward solving a national problem." This paper given in Buffalo, November 18, was published in Public Health Reports under the same date and we have read it with unusual attention. Concerning the article we would observe that while it makes plain the fact that the author's motive is fine and high—namely, the prevention of disease, the preservation of health and life—it makes it equally clear that the writer's method is faulty, even such, according to our estimate, as should not appear in a scientific paper or emanate from a man of scientific training.

This criticism is predicated upon the assumption that the subject of the paper is comparatively new; hence what we do not know about it is of quite enormous proportions. The need of moderation in statement is more than usual, because of the well known variability of symptoms of disease and, with greater particularity, of symptoms of nervous disease.

In a case then where such extreme moderation of statement is demanded we read, "The disease is certainly due to a specific microörganism which can be quite readily destroyed by the usual methods of disinfection." Again, "it has been demonstrated that the specific causative organism is of minute size." This language is positive and precise; it is either true or false; and it may be said of the statements that they are of enormous importance, if true. However, we are not aware of the announcement of even the first step of the demonstration of the germ origin of the neurotoxin of so-called poliomyelitis. Moreover, we regret the superficiality of the thought of the writer's statement that "virus" and "germ" are synonymous terms. We do not forget that the number of the toxins produced directly or indirectly by disease germs is large and is growing daily; but we also remember that strychin, abrin, crotin, ricin, robin, and the venins are dangerous poisons, many of them neurotoxins; and we protest against the dogmatic assumption of the writer, that where there is a virus there must be a germ, or that where there is a germ there must be a virus.

We think the writer is unfortunate in his references in that he fails to include the valuable contributions of Harbitz and Schule of the Pathological Institute of the University of Christiana, 1907; of Byron Bramwell, *Scottish Med. and Surg. Jour.*, June, 1908; of Buzzard, Barnes, James Miller, Perkins, Dudgeon, and R. Miller, in *Brain*, June, 1907. We consider these contributions of distinct importance, particularly as to the pathology,—that is, as to what is the so-called poliomyelitis.

We regret this stricture of a paper, the motive of which is commendable, while most of its expressions are beyond criticism. The prominence of the paper and the singular importance of the subject are our reasons.

